# Epigallocatechin-3-gallate (EGCG) inhibits imiquimod-induced psoriasis-like inflammation of BALB/c mice

**DOI:** 10.1186/s12906-016-1325-4

**Published:** 2016-08-31

**Authors:** Shuangshuang Zhang, Xiangdong Liu, Lihong Mei, Hongfeng Wang, Fang Fang

**Affiliations:** 1 Department of Dermatology, Jinshan Hospital, Fudan University, 1508 Longhang Road, Jinshan District, Shanghai, 201508 China; 2 Department of Plastic Surgery, Shanghai Jiao Tong University Affiliated Sixth People’s Hospital, Shanghai, China

**Keywords:** Antioxidant, EGCG, Imiquimod, Inflammation, Psoriasis

## Abstract

**Background:**

Psoriasis is a chronic inflammatory immune disease with undefined pathogenesis. It is associated with T cells, and the IL-23/IL17 axis is believed to be crucial in the pathogenesis. The present treatments have side effects that influence the compliance of patients. Tea polyphenol is extracted from tea polyphenols, and its main active ingredient is Epigallocatechin-3-gallate (EGCG), which has been shown to have antioxidant, anti-tumor, and anti-ultraviolet radiation effects. Here, we aim to report that EGCG can inhibit imiquimod (IMQ)-induced psoriasis-like inflammation.

**Methods:**

We used BALB/c mice, which were topically treated with IMQ for 6 consecutive days, as a psoriasis mouse model. Topical application of EGCG and treatment with EGCG were conducted in the experiments. Then observed the effects of the two methods on psoriasis-like mice dermatitis. Statistics are presented as the means ± standard error of mean (SEM) and compared using unpaired two-tailed Student’s t tests or one-way ANOVA.

**Results:**

Topical application of EGCG alleviated psoriasiform dermatitis, improved the skin pathological structure by reduce the expression of epidermal PCNA, promoted the expression of caspase-14. Treatment with EGCG attenuated skin inflammation, accompanied by reduced infiltrations of T cells; reduced percentages of CD11c^+^ DC in the composition of immunocytes of spleens; reduced levels of interleukin (IL)-17A, IL-17F, IL-22, IL-23 and malondialdehyde (MDA) in plasma; increased percentages of CD4^+^ T cells in the composition of immunocytes of spleens; and increased bioactivities of superoxide dismutase (SOD) and catalase (CAT) in plasma.

**Conclusions:**

All the results demonstrated that EGCG had anti-inflammatory, immune regulatory and antioxidant effects. It is a promising intervention in psoriasis in the future.

## Background

Psoriasis, which is a T-cell-mediated chronic inflammatory immune disease, is characterized by hyperproliferation and poor differentiation of epidermal keratinocytes and massive infiltration of leukocytes. The histological changes include (1) a thickened epidermis from epidermal hyperplasia and aberrant differentiation, (2) a reduced or absent granular layer, (3) a dermal inflammatory infiltrate, (4) increased dermal vascularity [1, 2]. It affects ≤2 % of the population of Northern European countries and 0.1 % ~0.3 % of the Asian population, and its accurate etiology is not well established [3, 4]. Although the pathogenesis of psoriasis is not fully understood, evidence suggests that many cytokines, including IL-6, IL-17A, IL17F, IL-22, IL-23 and TNF-α, are involved and interact as a network in the pathogenesis of psoriasis [3, 5–10]. Moreover, T cells, epidermal keratinocytes, dendritic cells (DCs), neutrophils, endothelial cells and fibroblasts play an important role in the development and maintenance of the disease [11].

Currently, the main therapies of psoriasis include psoralen and ultraviolet A (PUVA) photochemotherapy, topical treatment with steroids, retinoid, immunosuppressants, derivatives of vitamin D3, and biological therapies [12–15]. However, most of the therapies have various degrees of side effects and require a long duration of administration. Thus, it is urgent to develop another drug that is effective and does not induce side effects. EGCG is a possible solution. The literature has indicated anti-inflammatory, anti-tumor, antioxidant and anti-ultraviolet radiation effects of EGCG. For example, polyphenol-60, also known as green tea catechin compound, can down-regulate inflammatory cytokines such as IL-8, which has a therapeutic effect on acne [16]. EGCG has the function to promote DNA repair and inhibit inflammasome secretion, which is beneficial in the treatment of skin tumors [17, 18]. Katiyar et al. confirmed that the application of TP could significantly reduce the production of reactive oxygen species (ROS) in skin exposed to ultraviolet light, thereby inhibiting oxidative stress and oxidative damage [19]. Additionally, researchers have found that EGCG can reduce the risk of cancer caused by PUVA therapy, promote the normal differentiation of epidermal keratinocytes and function as an antiangiogenic compound [20–22]. Therefore, we suspect that EGCG has potential value in the treatment of psoriasis. There is no research on the other mechanisms of EGCG used in the treatment of psoriasis. Our research lays a solid foundation for the treatment of psoriasis with EGCG.

Imiquimod (IMQ) is an agonist of toll-like receptor-7/8 (TLR-7/8), which is widely used to treat condyloma acuminata, solar keratosis and basal cell carcinoma. Topical treatment with IMQ in mice can induce psoriasis-like skin inflammation through the interleukin (IL)-23/IL-17 axis, which provides a perfect mouse model of psoriasis [23]. This study is designed to investigate the effects of EGCG on IMQ-induced psoriasis-like BALB/c mice. We focused on the change in epidermal hyperplasia induced by EGCG, dermal infiltrates of T cells, anti-oxidation, key cytokines including IL-17A, IL-17 F, IL-22 and IL-23, and the composition of immunocytes in the spleen.

## Methods

### Mice and treatments

Six to eight week old BALB/c mice with an average weight of 20–25 g were purchased from the JieSijie Experimental Animal Company (Shanghai, China) and randomly divided into 7 groups (*N* = 6). The experiment is divided into two parts, the first part has three groups mice, is the external experimental group; the second part has four groups mice, is the gavage experimental group. The mice were housed under specific pathogen-free conditions and were fed with formal forage and water ad libitum in the animal experimental center of Jinshan Hospital. All experiments were conducted according to the principles of Guide for the Care and Use of Laboratory Animals in China and were approved by the animal ethics committee of Jinshan Hospital.

Firstly, an area of 5 cm × 4 cm was shaved from the backs of the three groups^,^ mice. The early intervention group mice (IMQ +6 EGCG) were applied IMQ and EGCG glycerin solvent (3 %, 30 mg/ml, glycerin solvent: 50 % glycerin, 50 % normal saline) for 6 consecutive days; medium-term intervention group mice (IMQ +3 EGCG) were firstly applied IMQ for 3 consecutive days, then IMQ and EGCG glycerin solvent for 3 consecutive days; negative control group mice were applied only IMQ cream for 6 consecutive days. (EGCG: PHR1333-1 g, Sigma-Aldrich, USA; IMQ: 0.25 g: 12.5 mg; Sichuan Med-Shine Pharmaceutical Company, LTD, China; glycerin: Sigma-Aldrich, USA) Photos were taken to observe the changes in the local skin. Then, all the three groups^,^ mice were killed, and samples of skin were collected for future experiments.

Secondly, the other four groups^,^ mice were conducted as follows. The normal (N) group and the control (C) group were applied normal saline (NS) by gavage twice a day for 3 weeks in a dosage of 0.2 ml/10 g/d. The high-dose (H) group and the low-dose (L) group were applied EGCG by gavage twice a day for 3 weeks in a dosage of 300 mg/kg/d and 150 mg/kg/d, respectively. After 3 weeks, an area of 5 cm × 4 cm was shaved from the backs of all the mice. The control group, the high-dose group and the low-dose group were applied 42 mg IMQ cream topically for 6 consecutive days. The normal group was applied simple IMQ base cream (Shanghai Dermatology Hospital, China) topically for 6 consecutive days. Photos were taken on the last day to observe the changes in the local skin. At the end-point of the experiment, weight was measured. Then, all the four groups^,^ mice were killed, and samples of blood, skin and spleen were collected for future experiments.

### Scoring severity of skin inflammation

To score the severity of inflammation of the back skin, an objective scoring system was developed based on the clinical Psoriasis Area and Severity Index (PASI). Erythema, scales and infiltration were scored independently on a scale from 0 to 4: 0, none; 1, slight; 2, moderate; 3, marked; 4, very marked. All scores were evaluated by two independent personnel. The cumulative score (erythema plus scaling plus infiltration) served to indicate the severity of inflammation (scale 0–12) [23].

### Changes of spleen in mice

The four gavage groups^,^ mice were weighed before being sacrificed, and then, each spleen was weighed. We calculated the spleen index (spleen index-spleen weight/ body weight) to reflect the dynamic body changes of the mice.

### Histopathological and immunohistochemical examinations

The skin samples from back lesions from each mouse were fixed in 4 % paraformaldehyde and embedded in paraffin for 24–36 hours. Sections (thickness-4 μm) were stained using hematoxylin and eosin (H&E). The tissue pathology was observed under a light microscope (Olympus BX43, Tokyo, Japan). Additionally, the thickness of the epidermis was measured using the measurement system of a microscope. For immunohistochemistry, the paraffin sections were stained with primary antibody: the three topical application of EGCG groups^,^ mice used rabbit anti-mouse PCNA antibody (KG22625, Kaiji, Jiangsu, China) and rabbit anti-mouse caspase-14 antibody (PA2104, Boster, CA); the four treatment with EGCG groups^,^ mice used rabbit anti-mouse CD3 polyclonal Ab (ab16044, Abcam, Cambridge, MA, USA). Then anti-ribbit secondary antibody (KIT9922, Maixin Bio, Fujian, China) were used. A brown color in epidermis or dermis was considered as positive staining. The evaluation of IHC was done by two independent personnel.

### Flow cytometry assays of lymphocytes isolated from mouse spleens

The collected mouse spleens from the four groups were weighed on an electronic balance and then grinded via 200 mesh screen to obtain single spleen cell suspension. The spleen cell suspension was lysed by RBC Lysis Buffer. Then, the cells (300–400 g) were spun at 4 °C, and the pellets were suspended in the appropriate PBS. For each staining, 1-2 × 10^9^ cells were fluorescently labeled after incubation in a dark room for 30 min at 4 °C with the following antibodies (all from Ebioscience, San Diego, CA, USA): anti-mouse CD3e FITC (no. 11-0031), anti-mouse CD4 APC (no. 17-0041), anti-mouse CD8a PE (no. 12-0081), and anti-mouse CD11c PE-Cyanine7 (no. 25-0114). All samples were detected on a flow cytometer (FACSCalibur; BD Biosciences, CA, USA) and analyzed with FlowJo ver. 7.6 software.

### Assess the levels of inflammatory cytokines including IL-17A, IL-17 F, IL-22 and IL-23 in plasma

Blood samples were collected from the heart of the four groups^,^ mice, and plasma was separated. Then, the separated plasma was spun at 10000 rpm for 5 minutes at room temperature, and we aspirated the supernatant. IL-17A, IL17F, IL-22 and IL-23 levels were assayed using a Mouse Th17 Milliplex Map Kit (Millipore Corporation, Billerica, MA, USA). Measurements were performed using the Bio-Plex MAGPIX Multiplex Reader (Bio-Rad Laboratories, Hercules, CA, USA).

### Activities of SOD, CAT and content of MDA in plasma

Blood samples were collected from the heart of the four groups^,^ mice and kept in tubes containing heparin sodium for 30 min at room temperature. Then, plasma was separated in a centrifuge (Backman Allegra X-22R Centrifuge, USA) at 2000 rpm for 20 minutes at room temperature. The assays of plasma superoxide dismutase (SOD), catalase (CAT) and malondialdehyde (MDA) were performed strictly according to the protocol with colorimetric Diagnostic Reagent Kit from Nanjing Jiancheng Bioengineering Institute (Nanjing, China).

### Statistical methods

Statistics are presented as the means ± standard error of mean (SEM) and compared using unpaired two-tailed Student’s t tests or one-way ANOVA. The results were analyzed using SPSS ver. 22.0 software. *P* < 0.05 was considered to be statistically significant. Pictures were produced using Photoshop ver.13.0 software and Graph Pad Prism5 software.

## Results

### EGCG treatment reduces IMQ-induced psoriasiform lesions of mice

**Fig. 1:**
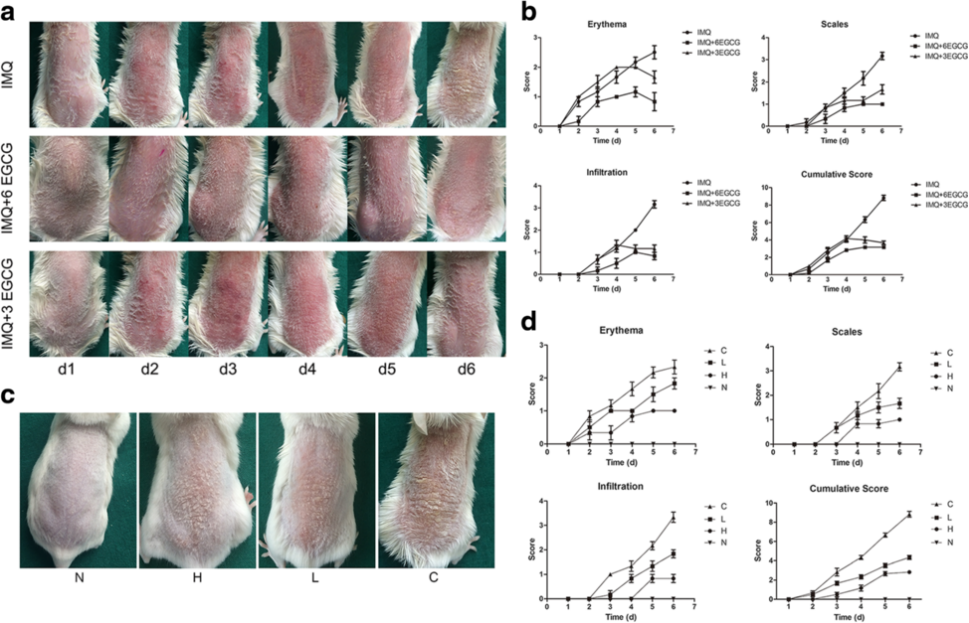
IMQ-induced psoriasis-like skin inflammation on the backs of BALB/c mice. (**a**) The back skin of the three groups displayed different grades of erythema, scales and infiltration. Photos were taken every day, d1 means one day after application of drugs and so on. (**b**) Erythema, scales and infiltration of the skin were identified from the first day on. The cumulative score (erythema plus scaling plus infiltration) was calculated (*n* = 6, Mean ± SEM). (**c**) The back skin of the four groups displayed different grades of erythema, scales and infiltration. Photos were taken after the application of IMQ for 6 days consecutively. (**d**) Erythema, scales and infiltration of the skin were identified from the first day on. IMQ, imiquimod; N, the normal group; H, the high-dose group; L, the low-dose group; C, the control group

First part: the external experimental group (the three groups):

It showed that the negative control group (treated with IMQ only) mice showed erythema, scales and infiltration from second day on and became more and more obvious. After 6 days, the mice skin of control group presented the performance similar to human plaque psoriasis. But in the early intervention group (IMQ +6 EGCG), the treated skin showed only a few erythema and thin scales, no obvious infiltration. The medium-term intervention group (IMQ +3 EGCG) mice had erythema, scales and infiltration from day two, but after application of EGCG, the performance were gradually relieved (Fig. 1a). We evaluated the severity of lesions with the Psoriasis Area and Severity Index (PASI), which was adjusted according to the extent of erythema, scales and infiltration from grade 1–5 in correspondence to 0–4 scores[23]. We can clearly see the changes of the skin from the PASI curve (Fig. 1b).

Second part: the gavage experimental group (the four groups):

The N group had no observable difference from the previous group. The C group had obvious erythema, scales and infiltration, whereas the H group and the L group showed that these features were less severe, especially in the H group (Fig. 1c). The mice in the C group had erythema, scales and infiltration on the back skin from day 2 onward, which became the most obvious on day 6. However, the H group showed a clear trend of relief of the lesions. The L group had a moderate tendency to present with erythema, scales and infiltration (Fig. 1d). The accumulative score of the H and L group was lower than that of the C group.

### EGCG attenuates the IMQ-induced histological changes of skin

**Fig. 2:**
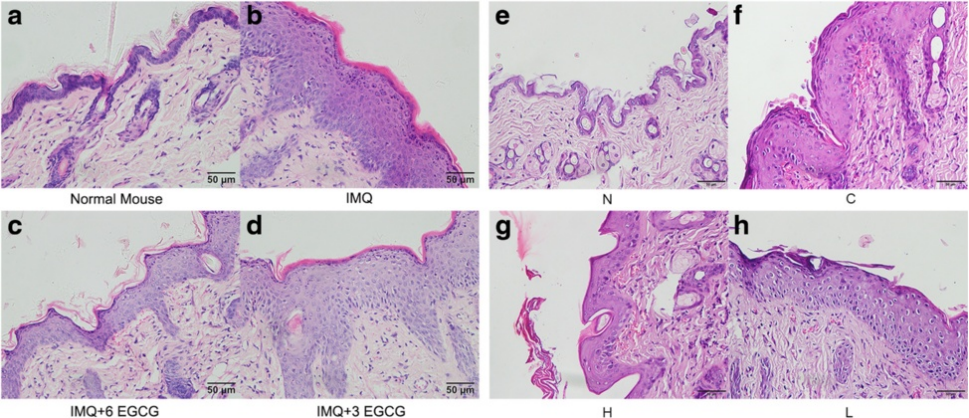
Respective histopathological changes of each group (H&E staining, original magnification × 400). Scale bar is 50 μm. (**a**–**d**) The control group mice were given IMQ only, and obvious parakeratosis, acanthosis and inflammatory cell infiltration were observed. The changes in the early intervention group (IMQ + 6EGCG) and medium-term intervention group (IMQ +3 EGCG) skin were eased, especially in the medium-term intervention group. (**e**–**h**)The N group mice received base cream for 6 days, and the epidermis was very thin. The C group mice were given IMQ instead, and obvious parakeratosis, acanthosis and inflammatory cell infiltration were observed. The H and L group mice were provided EGCG for 3 weeks before given IMQ. The changes in skin were eased compared with the C group, especially in the H group. EGCG, Epigallocatechin-3-gallate; IMQ, imiquimod; N, the normal group; H, the high-dose group; L, the low-dose group; C, the control group

First part: the external experimental group (the three groups):

Skin samples were fixed and stained using a standard hematoxylin and eosin (H&E) method. It was shown in Fig. 2a–d that the histological changes of the control group mice exhibited extensive psoriasiform lesions. We can see marked hyperkeratosis, parakeratosis, modest acanthosis, elongated rete ridges, and modest dermal inflammation. But these changes were significantly alleviated in the experimental groups, especially in the early intervention group. The thickness of the epidermis in the control group was 70–150 μm. The value significantly decreased in the experimental groups, which was 30–80 μm in the early intervention group and 60–95 μm in the medium-term intervention group.

Second part: the gavage experimental group (the four groups):

Results from H&E-stained IMQ-induced psoriasiform lesions showed increased parakeratosis, acanthosis and inflammatory cell infiltration (Fig. 2f). EGCG significantly attenuated these changes but did not clear them completely compared with the N group (Fig. 2e, g, h). This effect was related to the dose of EGCG. The effect of the H group was better than that of the L group (Fig. 2g, h). The thickness of the epidermis in the C group was 65–145 μm compared with 6–18 μm in the N group. The value significantly decreased in the experimental groups, which was 30–75 μm in the H group and 50–105 μm in the L group.

### Expression of PCNA and caspase-14 in the epidermis of mice skin of the external experimental group

**Fig. 3:**
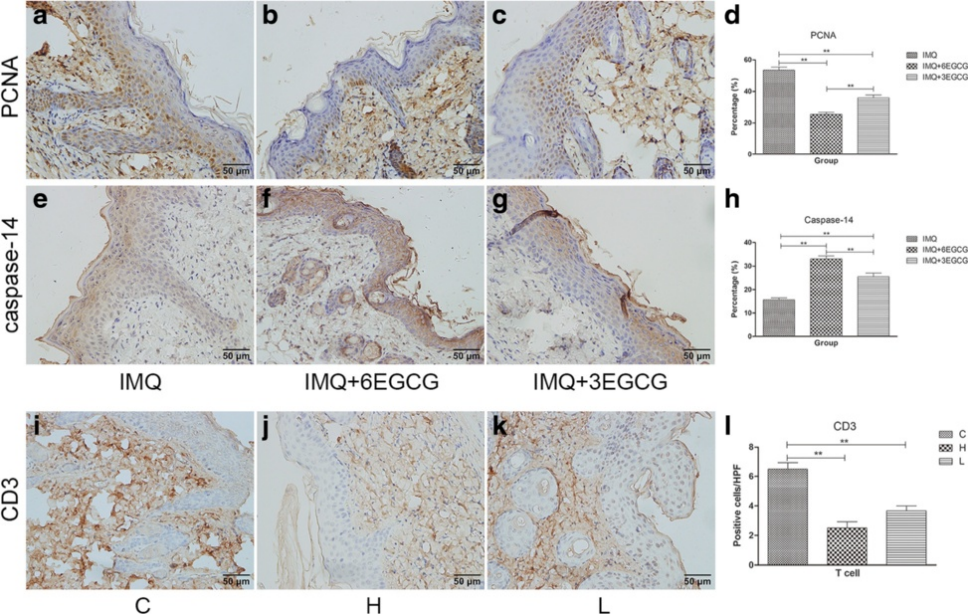
Expression of PCNA and caspase-14, infiltration of T cells were analyzed by immunohistochemistry (original magnification × 400). The numbers of positive cells were counted per high-power field (HPF) by two independent researchers. (**a**–**d**) The expression of PCNA was decreased in the early intervention group (IMQ + 6EGCG) and medium-term intervention group (IMQ +3 EGCG) compared with that in the control group. (**e**–**h**) The expression of caspase-14 was increased in the early intervention group (IMQ + 6EGCG) and medium-term intervention group (IMQ +3 EGCG) compared with that in the control group. (i- l) T cells in the dermal lesioned skin of the H and L group are significantly less than that in the C group (*n* = 6, Mean ± SEM, **P < 0.01). H, the high-dose group; L, the low-dose group; C, the control group

The control group mice skin exhibited PCNA immunostaining almost in all the epidermal layers (Fig. 3a), while PCNA immunostaining was found primarily in the basal layer of the early intervention group and the medium-term intervention group (Fig. 3b, c). The percentages of positive cells stained by PCNA in the three groups were statistically different (Fig. 3d, *P* < 0.01). Caspase-14 immunostaining in the early intervention group was obvious compared with that in the control group, and the medium-term intervention group showed modest staining (Fig. 3e–g). The percentages of positive cells stained by caspase-14 in the three groups were statistically different (Fig. 5h, *P* < 0.01).

### EGCG reduces infiltration of T cells in lesions of the gavage experimental group

T cells are essential in the pathogenesis of psoriasis. Therefore, we used immunohistochemistry to explore the effect of EGCG on the infiltration of T cells. In the control group, we can see the infiltration of T cells in the lesioned skin. As in the H and L group, the dermal infiltration of T cells was significantly reduced compared with the C group (Fig. 3i–l).

### Spleen index and the percentages of CD4^+^T cells, CD8^+^T cells and the DCs in spleens of the gavage experimental group

**Fig. 4:**
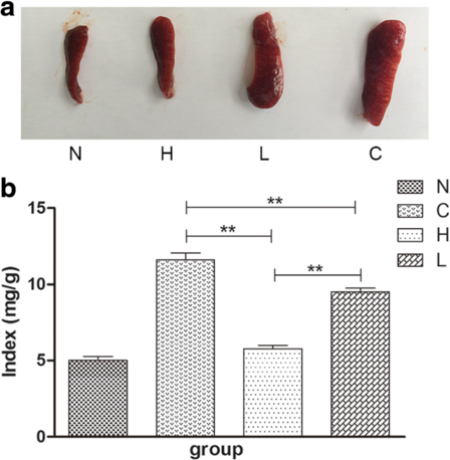
Changes in spleens of the four mice groups. (**a**) EGCG attenuated splenomegaly induced by topical IMQ. (**b**) The spleen index (spleen index = spleen weight/ body weight) was calculated (*n* = 6, Mean ± SEM, **P < 0.01). EGCG, Epigallocatechin-3-gallate; N, the normal group; H, the high-dose group; L, the low-dose group; C, the control group

A previous study showed that IMQ could induce splenomegaly through systemic effects [23]. We removed the spleens after topical application of IMQ on mice for 6 consecutive days. A similar result was seen in our study. The spleens of the C group were significantly bigger than those of the N group, whereas the spleens of the H and L group were smaller (Fig. 4a). This finding indicates that EGCG affects splenomegaly induced by IMQ. We further calculated the spleen index of each group. The spleen index of the H group was significantly lower than that of the C group and the L group. The index of the L group was also significantly lower than that of the C group (*P* < 0.01, Fig. 4b).

**Fig. 5:**
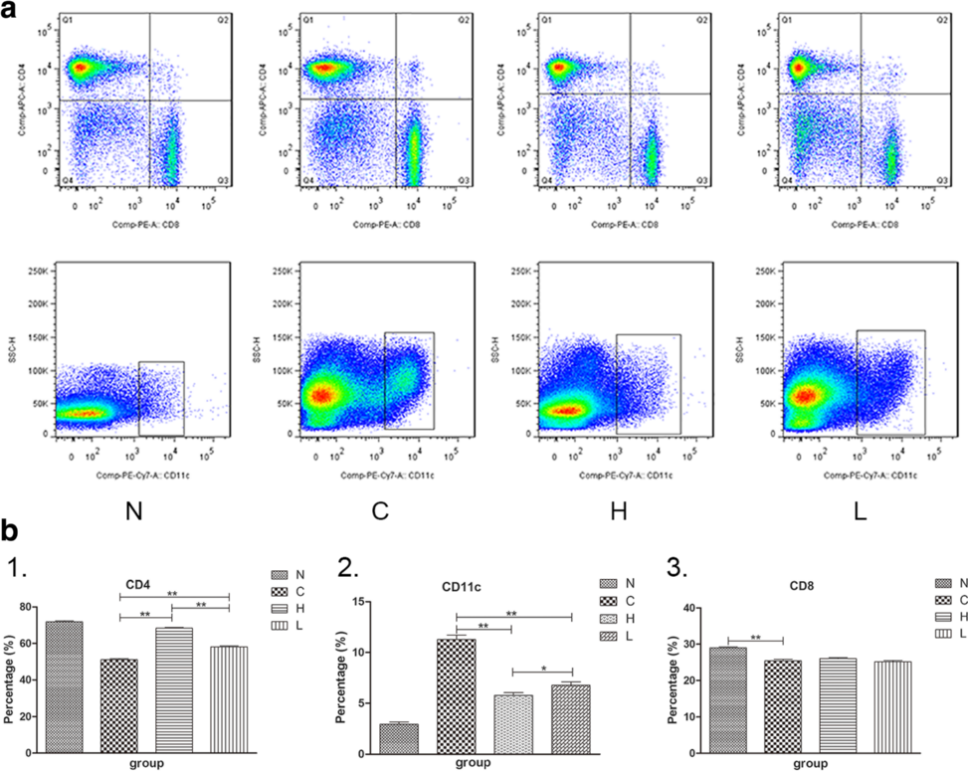
EGCG alters the percentages of CD4^+^T cells, CD8^+^T cells and CD11c^+^ dendritic cells in the spleens with the improvement of IMQ-induced psoriasis-like inflammation. (**a**) Flow cytometric analysis of CD4^+^T cells, CD8^+^T cells and CD11c^+^ dendritic cells in the spleens. (**b**1, **b**2) The percentages of CD4^+^T cells of the two experimental groups are increased, and the percentages of CD11c^+^ dendritic cells of the two groups are obviously reduced compared to the C group. The effect of the H group is better than that of the L group (*n* = 6, Mean ± SEM, *P < 0.05, ** *P* < 0.01). (**b**3) No significant difference in CD8^+^T cells of spleens between the H group, L group and C group (*n* = 6, Mean ± SEM, *P* > 0.05). EGCG, Epigallocatechin-3-gallate; IMQ, imiquimod; N, the normal group; H, the high-dose group; L, the low-dose group; C, the control group.

Psoriasis is an autoimmune inflammatory disease mediated by T cells and connected with DCs [24, 25]. Flow cytometric analysis revealed that the percentage of CD4^+^T cells in the C group was significantly decreased compared with that of the N group. EGCG effectively increased the percentage of CD4^+^T cells in spleens (*P* < 0.01, Fig. 5b1). In contrast, the percentage of CD11c^+^ DCs in the C group was significantly increased compared with the N group. EGCG reversed this effect (*P < 0.05, ** *P* < 0.01, Fig. 5b2). The percentage of CD8^+^T cells in the C group was significantly decreased compared with that of the N group. However, there was no significant difference between the H and L groups and the C group (*P* > 0.05, Fig. 5b3). These findings revealed that EGCG has no effects on CD8^+^T cells but affects immunocytes such as CD4^+^T cells and CD11c^+^ DCs.

### EGCG reduces the expression of inflammatory cytokines including IL-17A, IL-17 F, IL-22 and IL-23 of the gavage experimental group

**Fig. 6:**
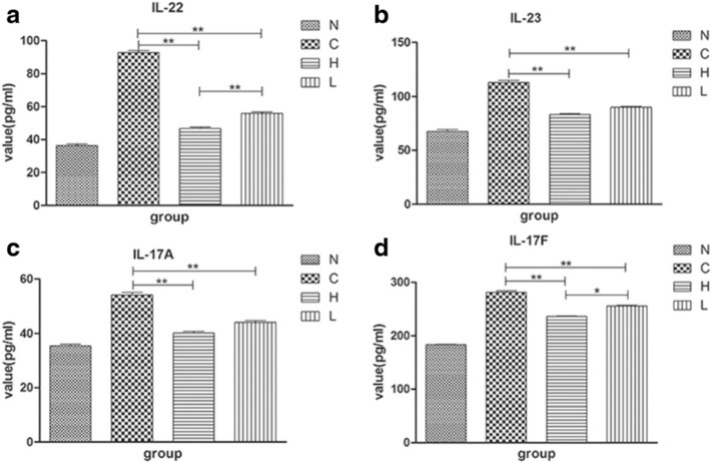
EGCG plays a role in the changes of inflammatory factors in plasma. (**a**, **d**) EGCG decreases the expression of interleukin (IL)-22 and IL-17 F in plasma, and these levels were more decreased in the H group than in the L group (*n* = 6, Mean ± SEM, * *P* < 0.05, **P < 0.01). (**b**, **c**) The relative quantities of IL-23 and IL-17A in the H and L groups are lower than in the C group (*n* = 6, Mean ± SEM, **P < 0.01). There is no significant difference between the H group and the L group (*n* = 6, Mean ± SEM, *P* > 0.05). EGCG, Epigallocatechin-3-gallate; N, the normal group; H, the high-dose group; L, the low-dose group; C, the control group.

We have stated that IMQ-induced psoriasis-like skin inflammation in mice is mediated via the IL-23/IL-17 axis. IL-23 secreted by dendritic cells can induce the proliferation of Th17 cells, which can activate and secrete IL-17A, IL-17 F, IL-22 and IL-23 [7, 9, 23]. The expressions of the four inflammatory cytokines in plasma were significantly higher in the C group than in the N group. However, in the H group and the L group, the expression was significantly lower than in the C group, especially in the H group (*P < 0.05, ** *P* < 0.01, Fig. 6). The results indicate that EGCG plays an effective role in reducing inflammatory factors to alleviate the IMQ-induced inflammatory effects.

### Bioactivities of SOD, CAT in plasma and the content of MDA in plasma of the gavage experimental group

**Fig. 7:**
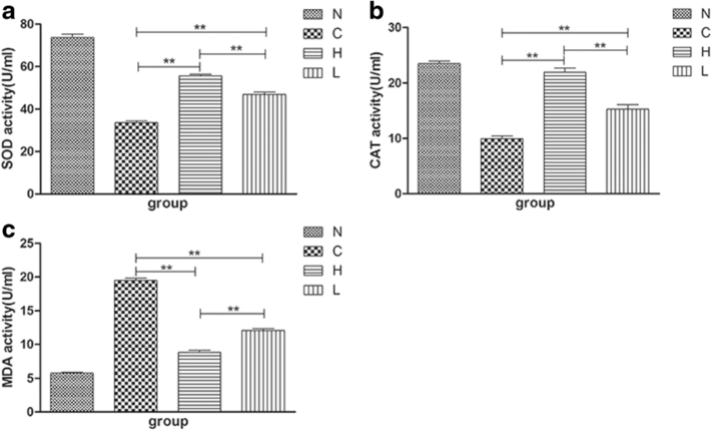
EGCG can balance the amount of pro-oxidants and antioxidants in plasma. (**a**, **b**, **c**) The plasma bioactivities of superoxide dismutase (SOD) and catalase (CAT) of the H and L groups were increased, and that of malondialdehyde (MDA) was decreased compared with the C group (*n* = 6, Mean ± SEM, **P < 0.01). EGCG, Epigallocatechin-3-gallate; IMQ, imiquimod; N, the normal group; H, the high-dose group; L, the low-dose group; C, the control group.

EGCG has been proven to have antioxidant function [26]. The anti-oxidases such as SOD and CAT can protect organisms from the damage of oxidative stress. Therefore, to explore the role of EGCG as an antioxidant in IMQ-induced psoriasiform changes, we investigated the activities of SOD and CAT in plasma and the content of MDA. Fig. 7a–c shows that the bioactivities of SOD and CAT of the C group were significantly reduced compared with those of the N group. However, the content of MDA of the C group was significantly increased. In contrast, the bioactivities of SOD and CAT of the H and L groups were significantly enhanced compared with those of the C group, and the content of MDA of the H and L groups was significantly decreased compared with that of the C group. The antioxidant effect of EGCG displayed a dosage effect. The H group demonstrated better results than the L group.

## Discussion

Only a few animal models can imitate the pathogenesis of human psoriasis. IMQ is a toll-like receptor agonist, which is used to treat condyloma acuminata, solar keratosis, basal cell carcinoma and other conditions [27, 28]. Van der Fits et al. revealed that IMQ-induced psoriasis-like inflammation markedly reflected the characteristics of plaque type psoriasis, which include hyperproliferative keratinocyte, parakeratosis, absence of a granular layer, and existence of inflammatory cells consisting of T cells, neutrophils, DCs and vascular proliferation [23]. According to the results, we also successfully created a mouse model of psoriasis with IMQ (Fig. 1a, c).

Numerous studies have demonstrated that EGCG has anti-inflammatory, anti-carcinogenic, antioxidant, anti-aging and anti-mutagenic effects [17, 29]. Stephen Hsu et al. found that the symptoms of flaky skin in mice were significantly reduced after taking a bath in water with 0.5 % green tea polyphenol (GTP) [30]. Therefore, we suspect that EGCG may have a therapeutic effect on psoriasis. In our study, we revealed that topical application or treat with EGCG attenuates IMQ-induced inflammation in BALB/c mice, improving its clinical manifestation and significantly decreasing the adjusted PASI scores consisting of erythema, scales and infiltration of the lesions (Fig. 1b, d), which provide further evidence of our conjecture.

Proliferating cell nuclear antigen (PCNA) is a 36 kDa nuclear non histone protein polypeptide, is one of the active marker of cell proliferation [31]. One of the basic features of psoriasis is abnormal proliferation of keratinocytes, and then thick scales form. The results showed that topical application of EGCG can reduce PCNA expression, effectively inhibit IMQ-induced abnormal proliferation of epidermal cells and relieve psoriasis-like appearance (Fig. 3a–d). Caspase-14 is a member of the caspase family, mainly distributed in the epidermis, is rare in other tissues, and its main function is to participate in the terminal differentiation of epidermal cells and normal skin barrier formation [32]. Obviously, the skin barrier of psoriasis is damaged, there is abnormal proliferation and differentiation of keratinocytes and parakeratosis is seen [31]. Our experiments of caspase-14 immunohistochemical staining in mice damaged epidermis confirmed the topical application of EGCG effectively increase the epidermal caspase-14 expression of IMQ-induced psoriasis-like lesions and early intervention had better effects (Fig. 3e–h). It meant that EGCG played a role in promoting the normal differentiation of psoriatic epidermis in mice.

Psoriasis is a T-cell-mediated chronic inflammatory immune skin disease [33]. The current study found that T cell is significantly elevated in psoriatic lesions. The cytokine network, which is activated by T cells, is an important component in the inflammatory response of psoriasis. This experiment found that EGCG can effectively reduce the expression of CD3 in the skin lesions of mice (Fig. 3i–l), which proved that the treatment effect of EGCG might reduce the expression of T cells in mice skin.

The spleen is an important organ in the immune system. IMQ induced splenomegaly is related to the systemic effects, whereas EGCG significantly reduced the spleen index, which was accompanied by the changes in cellular composition. The percentage of CD4^+^T cells in spleens was decreased in the C group mice compared with the N group (Fig. 5b1). CD11c^+^ dendritic cells correspond to interstitial dendritic cells in other tissues and represent the most abundant dendritic cell type in the dermis [34]. The percentage of CD11c^+^ dendritic cells was increased in the C group mice compared with the N group (Fig. 5b2). This phenomenon was reversed by EGCG in addition to the remission of IMQ-induced psoriasis-like inflammation (Fig. 5b1, 2). All these results suggest that EGCG may regulate the immune function of psoriasis-like mice through the regulation of CD4^+^T cells and CD11c^+^ dendritic cells. Although CD8^+^T cells are important participants in psoriasis, there was no significance in the proportion of CD8^+^T cells (Fig. 5b3). This finding may be the difference between mouse model and human psoriasis.

Recently, a group of CD4^+^T cells found in multiple inflammatory diseases and autoimmune diseases, which can secrete IL-17, has been named: Th17. IL-23 mainly comes from activated macrophages and dendritic cells, and it is the key cytokine in promoting the differentiation and development of Th17 cells [1]. Th17 cells and the cytokines secreted by them, such as IL-17A, IL-17 F, IL-22 and IL-21, play a role in numerous chronic inflammatory diseases including psoriasis [1, 34]. These Th17-related cytokines can further influence keratinocytes and other inflammatory cells in the skin, causing local inflammation and proliferation of keratinocytes [7]. All these findings confirmed the important role of the IL-23/IL-17 axis in the pathogenesis of psoriasis. As already mentioned, IMQ-induced psoriasis-like skin inflammation is related to the IL-23/IL-17 axis. The data shown in our study revealed that inflammatory cytokines in plasma including IL-17A, IL-17 F, IL-22 and IL-23 were reduced with the attenuation of IMQ-induced psoriasis-like inflammation in the H and L group mice (Fig. 6). However, the accurate mechanisms of EGCG in reducing these cytokines are undefined. However, we suspect that the anti-inflammatory effect of EGCG is through the IL-23/IL-17 axis.

The skin has a complex defense system to protect the body from harmful environmental and chemical substances, but excessive exposure can overwhelm the system, leading to oxidative stress and oxidative damage. During the energy-producing process of reducing molecular oxygen to water, reactive oxygen species (ROS) are formed. ROS are involved in the proliferation, differentiation, apoptosis, immune response and other issues. An over-production of ROS can take away numerous ROS-scavenging enzymes, including SOD and CAT [26]. This result is harmful to proteins, lipids, and DNA. Oxidative stress can also be assessed by the level of MDA in plasma [35]. Healthy skin maintains a dynamic balance between oxidation and antioxidation, and if this balance is destroyed, some diseases manifest, including psoriasis [36]. Previous studies have also found that antioxidants can improve the symptoms of psoriasis. Moreover, the antioxidant function of EGCG has been well established [37–39]. In our results, the bioactivities of SOD and CAT in the C group were much lower than that of the N group, whereas the content of MDA was higher than that of the N group (Fig. 7). After application of EGCG, the appearance reversed but did not reach the normal level. These results were consistent with the morphological and histological changes of the mice. The results indicate that EGCG can improve the symptoms of mice psoriasis by regulating antioxidant factors.

The topical application of EGCG and treatment of EGCG were used in our study. Moreover, two dose groups were used as the experimental group in our experiment to explore the different effects between high-dose EGCG and low-dose EGCG. The high-dose (H) group and the low-dose (L) group were given EGCG by gavage twice a day for 3 weeks at a dosage of 300 mg/kg/d and 150 mg/kg/d, respectively. Most of the data, including the clinical and pathological features of mice skin, spleen index, proportion of CD4^+^T cells and CD11c^+^ dendritic cells in the spleen, and levels of IL-22, IL-17 F, SOD, CAT and MDA in plasma, showed that the anti-inflammatory, immune regulatory and antioxidant effects of EGCG of the H group were better than those of the L group, and early intervention made better effects (Figs 1, 2, 3, 4, 5, 6 and 7). These findings indicate that these effects of EGCG may be dose dependent.

## Conclusion

In conclusion, we demonstrated that EGCG attenuates IMQ-induced psoriasis-like inflammation; reduces expression of PCNA; increases expression of caspase-14; reduces infiltrations of T cells in mice skin; regulates the percentages of CD4^+^T cells and CD11c^+^ DC of spleens; down-regulates the levels of IL-17A, IL17F, IL-22, IL-23 and malondialdehyde (MDA) in plasma; and increases bioactivities of superoxide dismutase (SOD) and catalase (CAT) in plasma to treat psoriasis. Moreover, EGCG is extracted from green tea, which is cheap and easy to obtain. EGCG is therefore a promising adjuvant intervention in treating psoriasis in the future.
